# Neurobiological underpinnings of reward anticipation and outcome evaluation in gambling disorder

**DOI:** 10.3389/fnbeh.2014.00100

**Published:** 2014-03-25

**Authors:** Jakob Linnet

**Affiliations:** ^1^Research Clinic on Gambling Disorders, Aarhus University HospitalAarhus, Denmark; ^2^Center of Functionally Integrative Neuroscience, Aarhus UniversityAarhus, Denmark; ^3^Division on Addiction, Cambridge Health AllianceCambridge, MA, USA; ^4^Department of Psychiatry, Harvard Medical School, Harvard UniversityCambridge, MA, USA

**Keywords:** anticipation, reward prediction error, reward prediction, incentive salience, dopamine, gambling disorder, pathological gambling

## Abstract

Gambling disorder is characterized by persistent and recurrent maladaptive gambling behavior, which leads to clinically significant impairment or distress. The disorder is associated with dysfunctions in the dopamine system. The dopamine system codes reward anticipation and outcome evaluation. Reward anticipation refers to dopaminergic activation prior to reward, while outcome evaluation refers to dopaminergic activation after reward. This article reviews evidence of dopaminergic dysfunctions in reward anticipation and outcome evaluation in gambling disorder from two vantage points: a model of reward prediction and reward prediction error by Wolfram Schultz et al. and a model of “wanting” and “liking” by Terry E. Robinson and Kent C. Berridge. Both models offer important insights on the study of dopaminergic dysfunctions in addiction, and implications for the study of dopaminergic dysfunctions in gambling disorder are suggested.

## Neurobiological underpinnings of reward anticipation and outcome evaluation in gambling disorder

Gambling disorder is characterized by persistent and recurrent maladaptive gambling behavior, which leads to clinically significant impairment or distress (American Psychiatric Association [DSM 5], 2013). Gambling disorder was recently reclassified from “pathological gambling” (an impulse control disorder) to a “behavioral addiction” under the substance use classification, which emphasizes the association between gambling disorder and other types of addiction.

Gambling disorder is associated with dysfunctions in the dopamine system. The dopamine system is sensitive to behavioral stimulation related to monetary reward, particularly in the ventral striatum (Koepp et al., 1998; Delgado et al., 2000; Breiter et al., 2001; de la Fuente-Fernández et al., 2002; Zald et al., 2004). Dopaminergic dysfunctions in the ventral striatum are linked to gambling disorder (Reuter et al., 2005; Abler et al., 2006; Linnet et al., 2010, 2011a,b, 2012; van Holst et al., 2012; Linnet, 2013).

The dopamine system codes *reward anticipation* and *outcome evaluation*. Reward anticipation refers to dopaminergic activation prior to reward, while outcome evaluation refers to dopaminergic activation after the reward. This article reviews evidence on dopaminergic dysfunctions in reward anticipation and outcome evaluation in gambling disorder from two vantage points: a model of reward prediction and reward prediction error by Schultz et al. (Fiorillo et al., 2003; Schultz, 2006; Tobler et al., 2007; Schultz et al., 2008), and a model of “wanting” and “linking” by Robinson and Berridge (Robinson and Berridge, 1993, 2000, 2003, 2008; Berridge and Aldridge, 2008; Berridge et al., 2009). It is suggested that gambling disorder may provide a “model disorder” of addiction for the two approaches, which is not confounded by ingestion of exogenous substances.

The ventral striatum and the nucleus accumbens (NAcc) play a central role in both models, which is consistent with findings of dopamine dysfunctions in the ventral striatum in gambling disorder. Therefore, this review focuses on the ventral striatum in relation to gambling disorder. Other relevant areas include the prefrontal cortex (e.g., orbitofrontal cortex) and other areas of the basal ganglia (e.g., the putamen, nucleus or caudate).

## Reward prediction and reward prediction error

Reward prediction refers to the anticipation of reward, while reward prediction error refers to the outcome evaluation. Reward prediction and reward prediction error are associated with the learning of reward properties of stimuli. According to Wolfram Schultz (2006), reward prediction and reward prediction error derive from Kamin’s *blocking rule* (Kamin, 1969), which suggests that a reward that is fully predicted does not contribute to learning. A stimulus that can be entirely predicted contains no new information, and the reward prediction error rate is therefore zero. Rescola and Wagner described the so-called *Rescola-Wagner learning rule* (Rescola and Wagner, 1972), which states that learning slows progressively as the reinforcer becomes more predicted.

In random binary outcome conditions, e.g., reward vs. no-reward, the *expected value* (EV) is the average value that can be expected from a given stimulus, which is a linear function of reward probability. In contrast, *uncertainty*, which can be defined as the variance (σ^2^) of a probability distribution (Schultz et al., 2008), is the mean squared deviation from the EV, which is an inverse U-shaped function. Midbrain and striatal dopamine coding of EV and uncertainty follow linear and quadratic functions of reward prediction similar to their mathematical expressions (Fiorillo et al., 2003; Preuschoff et al., 2006; Schultz, 2006). The dopamine system also codes deviations in outcome from the reward prediction, i.e., reward prediction error: “…dopamine neurons emit a positive signal (activation) when an appetitive event is better than predicted, no signal (no change in activity) when an appetitive event occurs as predicted, and a negative signal (decreased activity) when an appetitive event is worse than predicted…[and] dopamine neurons show bidirectional coding of reward prediction errors, following the equation Dopamine response = Reward occurred−Reward predicted” (Schultz, 2006, pp. 99–100).

Fiorillo et al. (2003) investigated dopamine activation in reward prediction and reward prediction error in relation to EV and uncertainty (i.e., variance in outcome). In the study, two monkeys were exposed to stimuli with varying reward probabilities (*P* = 0, *P* = 0.25, *P* = 0.5, *P* = 0.75 and *P* = 1.0). The rate of anticipatory licking and the activation of dopamine neurons in the ventral midbrain (area A8, A9 and A10) were recorded. Dopaminergic coding of reward prediction was measured as a *phasic* signal immediately after stimulus presentation, while coding of reward prediction error was measured as a phasic signal immediately after the outcome of the stimulus (reward or no reward). Dopaminergic coding of uncertainty was measured as a *sustained* signal from stimulus presentation to outcome.

The authors reported three main results. First, the reward probabilities of stimuli were correlated with the anticipatory licking rate and the anticipatory phasic dopamine response. This suggests that the reward probability reinforced the dopaminergic activation and the behavioral response. Second, the sustained dopamine response toward uncertainty followed the properties of variance, i.e., it was largest toward stimuli with 50% reward probability (*P* = 0.5), smaller toward stimuli with *P* = 0.75 and *P* = 0.25, and smallest toward stimuli with *P* = 1.0 and *P* = 0.0. Third, rewarded stimuli with lower reward probability had a larger phasic dopamine response following the reward, which suggests a larger positive reward prediction error signal; rewarded stimuli with higher reward probability had a smaller phasic dopamine response following the reward, which suggests a smaller reward prediction error signal.

Neurobiological studies of gambling in humans support the evidence of reward prediction and reward prediction error. Abler et al. (2006) used functional magnetic resonance imaging (fMRI) to investigate reward prediction and reward prediction error in an incentive task where participants were shown five figures associated with different reward probabilities (*P* = 0.0, *P* = 0.25, *P* = 0.50, *P* = 0.75, and *P* = 1.0). The results showed a significant anticipatory blood oxygen level dependent (BOLD) activation in the NAcc, which was proportional to the reward probability. Furthermore, there was a significant interaction between outcome and BOLD activation in the NAcc, where the BOLD activation was higher when low probability stimuli were rewarded, and lower when high probability stimuli were rewarded.

Preuschoff et al. (2006) used a card guessing task to investigate the relationship between risk and uncertainty in relation to anticipated reward. The task consisted of 10 cards ranging from 1 to 10, where two cards were drawn in succession. Before the drawing of the second card participants had to guess whether the first card would be higher or lower than the second card. The results showed that reward probability was linearly associated with immediate BOLD activation: higher reward probability was associated with a higher immediate anticipatory BOLD signal, and lower reward probability was associated with a lower immediate anticipatory BOLD signal. In contrast, uncertainty showed an inverse U-shaped relation with late BOLD activation: the highest anticipatory BOLD signals were seen around maximum uncertainty (*P* = 0.5) and the lowest anticipatory BOLD signals were seen around maximum certainty (*P* = 1.0 and *P* = 0.0).

Neurobiological studies support the notion of dopaminergic dysfunctions of reward anticipation in gambling disorder. van Holst et al. (2012) compared 15 gambling disorder sufferers with 16 healthy controls in a fMRI study investigating reward anticipation in a card guessing task. Gambling disorder sufferers showed a significant increase in BOLD activation in the bilateral ventral striatum and in the left orbitofrontal cortex toward gain-related EV. This suggests an increased BOLD activation toward reward anticipation. No differences in BOLD activation were found toward outcome evaluation. Linnet et al. (2012) compared 18 gambling disorder sufferers and 16 healthy controls in a positron emission tomography (PET) study using the Iowa Gambling Task (IGT). Dopamine release in the striatum of gambling disorder sufferers showed a significant inverted U-curve with the probability of advantageous IGT performance. Gambling disorder sufferers with maximum uncertainty of outcome (*P* = 0.5) had a larger dopamine release than individuals with IGT performance closer to certain gains (*P* = 1.0) or certain losses (*P* = 0.0). This is consistent with the notion of dopaminergic coding of uncertainty. No interaction was found between dopamine release and uncertainty among healthy control subjects, which could suggest a stronger reinforcement of gambling behavior among gambling disorder sufferers. Therefore, in gambling disorder dopaminergic anticipation of reward and uncertainty might represent a dysfunctional reward anticipation, which reinforces the gambling behavior despite losses.

In outcome evaluation the evidence suggests a blunted dopamine response in gambling disorder sufferers. Reuter et al. (2005) compared 12 gambling disorder sufferers with 12 healthy controls in a card guessing task. Gambling disorder sufferers showed a significantly lower BOLD response in the ventral striatum toward winning compared with healthy controls. Furthermore, gambling disorder sufferers showed a significant negative correlation between the BOLD activation and severity in gambling symptoms, which suggests a blunted outcome evaluation in gambling disorder.

One of the limitations of the reward prediction and reward prediction error model is that it is not a theory of addiction or gambling disorder, *per se.* In other words, while the increased dopaminergic activation toward uncertainty might be a central mechanism in the reinforcement of gambling behavior, it does not explain why some individuals become addicted to gambling, while others do not. In contrast, the incentive-sensitization model suggests that addictive behavior is associated with a combination of dopaminergic reinforcement and changes to the dopamine system (sensitization) following repeated drug exposure.

## Incentive-sensitization model of “wanting” and “liking”

Terry E. Robinson and Kent C. Berridge (Robinson and Berridge, 1993, 2000, 2003, 2008; Berridge and Aldridge, 2008; Berridge et al., 2009) have proposed an *incentive-sensitization* model, which distinguishes pleasure (“liking”) from incentive salience (“wanting”) in addiction. “Wanting” is associated with anticipation of reward, while “liking” is associated with outcome evaluation.

The incentive-sensitization model focuses on the dopamine system as a core neurobiological basis of addiction. The ventral striatum and its main component the NAcc are associated with addiction. Changes in the dopamine system associated with drug exposure render the brain circuits hypersensitive or “sensitized” to drugs or drug cues. Sensitization from repeated drug exposure may also occur at the level of psychomotor or locomotor activity. Sensitization is linked with increased incentive salience, which is the cognitive process associated with drug seeking and drug taking behavior. Incentive salience (“wanting”) refers to a motivational state, which can be conscious or unconscious, goal-oriented or non goal-oriented, and pleasurable or non-pleasurable:

“The quotation marks around the term “wanting” serve as caveat to acknowledge that incentive salience means something different from the ordinary common language sense of the word wanting. For one thing, “wanting” in the incentive salience sense need not have a conscious goal or declarative target…. Incentive salience is separable from beliefs and declarative goals that constitute cognitive aspects of “wanting”” (Berridge and Aldridge, 2008, pp. 8–9).

Incentive salience (“wanting”) increases after repeated exposure to drugs and drug cues, while pleasure (“liking”) remains the same or decreases over time. The incentive-sensitization model of “wanting” and “liking” offers an explanation for the apparent paradox that individuals with substance use disorder have an increased desire for drugs despite getting less pleasure from taking them. Incentive “hotspots” have been identified in the NAcc: activation in the medial NAcc shell is distinctly associated with “liking”, while activation throughout the NAcc (particularly around the ventral pallidum) is associated with “wanting” (Berridge et al., 2009).

Incentive sensitization defines the relationship between incentive salience and sensitization. Incentive salience must be coupled with sensitization to account for addictive behavior: an increase in dopamine binding does not define incentive sensitization, but an increase in dopamine binding in relation to particular drug cues does; locomotor activity does not indicate incentive sensitization, but running around to get drugs does; psychomotor preoccupation does not indicate incentive sensitization, but an obsession with taking drugs does. Therefore, simple reinforcement of behavior is insufficient to account for addictive behavior.

“The central idea is that addictive drugs enduringly alter NAcc-related brain systems that mediate a basic incentive-motivational function, the attribution of incentive salience. As a consequence, these neural circuits may become enduringly hypersensitive (or “sensitized”) to specific drug effects and to drug-associated stimuli (via activation by S-S associations). The drug-induced brain change is called neural sensitization. We proposed that this leads psychologically to excessive attribution of incentive salience to drug-related representations, causing pathological “wanting” to take drugs” (Robinson and Berridge, 2003, p. 36).

Berridge and Aldridge (2008) provide an example of the incentive-sensitization approach to research in addiction. In this approach, animals are trained under two conditions: first, the animals are conditioned to work (press a lever) for rewards (e.g., food pellets), and must persist working to earn rewards. In a separate training session the animals receive rewards without having to work for them, where each reward is associated with an auditory tone cue for 10–30 s, which is the conditioned stimulus (CS+). After training, the animals are tested in an extinction paradigm where “wanting” is measured as the number of lever presses the animal is willing to perform without receiving a reward. Since the animals receive no rewards, the “wanting” is not confounded by consumption of reward. The key of the paradigm is to test changes in behavior when the conditioned auditory stimulus is introduced during different drug induced states. In a series of studies, Wyvell and Berridge (2000, 2001) showed that rats injected with amphetamine microinjections in the NAcc shell had significantly more lever presses when the conditioned auditory stimulus was introduced compared to rats injected with saline microinjections. In a related experiment, Wyvell and Berridge (2000, 2001) found that the measures of liking (facial reaction to receiving a sugar reward) did not differ whether the animals received saline or amphetamine microinjections. These findings suggest that amphetamine is associated with an increased cue-triggered “wanting”, but not with increased pleasure (“liking”) from receiving the reward.

The incentive-sensitization model’s suggestions of increased “wanting” and decreased “liking” in addiction are consistent with the findings from the gambling disorder literature of increased dopamine activation to anticipated reward (Fiorillo et al., 2003; Abler et al., 2006; Preuschoff et al., 2006; Linnet et al., 2011a, 2012) and blunted dopamine activation to outcome of reward (Reuter et al., 2005). These findings suggest that dopaminergic dysfunctions toward *anticipated* rewards, rather than actual rewards, reinforce gambling behavior among gambling disorder sufferers. The sensitization of the dopamine system toward anticipated rewards rather than incurred rewards can explain why gambling disorder sufferers continue gambling despite losses, and might play a central role in the formation of erroneous perceptions about the likelihood of winning from gambling (Benhsain et al., 2004).

One of the limitations of the incentive-sensitization model is that individuals with substance use disorder have lower dopamine release and lower dopamine receptor availability despite having increased incentive-sensitization:

“However, it must be acknowledged that the current literature contains conflicting results about brain dopamine changes in addicts. For example, it has been reported that detoxified cocaine addicts actually show a decrease in evoked dopamine release rather than the sensitized increase described above…. Another finding in humans that seems inconsistent with sensitization is that cocaine addicts are reported to have low levels of striatal dopamine D2 receptors even after long abstinence…. This suggests a hypodopaminergic state rather than a sensitized state” (Robinson and Berridge, 2008, p. 3140).

While lower binding potentials are reported in substance use disorders, there is no evidence of decreased binding potentials in the gambling disorder literature (Linnet, 2013). Therefore, gambling disorder might serve as a “model” disorder for the incentive-sensitization model, as gambling is not confounded by the ingestion of exogenous substances.

## Implications of reward anticipation and outcome evaluation in gambling disorder

The models by Schultz et al. and Robinson and Berridge provide important insights on the study on gambling disorder. The reward prediction and reward prediction error model by Schultz et al. offers an explanation for the behavioral reinforcement of reward anticipation in addiction, while the incentive-sensitization model by Robinson and Berridge explains the mechanisms of “wanting” and “liking” in addiction. At the same time, gambling disorder may serve as a “model” disorder in addressing certain aspects of the two models.

First, the lower levels of binding potentials reported in substance use disorder are not seen in gambling disorder (Linnet et al., 2010, 2011a,b, 2012; Clark et al., 2012; Boileau et al., 2013). This might suggest that incentive sensitization can occur independently of baseline dopamine binding in support of the incentive-sensitization model.

Second, while the studies by Fiorillo et al. (2003) and Preuschoff et al. (2006) support the notion of sustained anticipatory dopamine activation toward uncertainty, more research is needed to determine whether or not this mechanism is associated with dopaminergic dysfunctions in gambling disorder.

Third, the gambling disorder literature suggests increased brain activation toward reward anticipation and blunted activation toward outcome evaluation. This is consistent with the incentive-sensitization model’s suggestion of increased “wanting” but decreased “liking” in addiction and the notion of sustained anticipatory dopamine activation in reward prediction. Dopaminergic dysfunction in reward anticipation might constitute a common mechanism of addiction, because it occurs in the absence of reward. Therefore, reward anticipation may have a similar (dys)function, whether the reward is food, drugs or gambling. Further studies should address reward anticipation and outcome evaluation in gambling disorder.

## Conflict of interest statement

The author declares that the research was conducted in the absence of any commercial or financial relationships that could be construed as a potential conflict of interest.
